# Computational Insights into the Deleterious Impacts of Missense Variants on *N*-Acetyl-d-glucosamine Kinase Structure and Function

**DOI:** 10.3390/ijms22158048

**Published:** 2021-07-28

**Authors:** Raju Dash, Sarmistha Mitra, Yeasmin Akter Munni, Ho Jin Choi, Md. Chayan Ali, Largess Barua, Tae Jung Jang, Il Soo Moon

**Affiliations:** 1 Department of Anatomy, Dongguk University College of Medicine, Gyeongju 38066, Korea; rajudash.bgctub@gmail.com (R.D.); sarmisthacu@gmail.com (S.M.); yeasminakteracce@gmail.com (Y.A.M.); chjack@naver.com (H.J.C.); 2 Department of Biotechnology & Genetic Engineering, Faculty of Biological Sciences, Islamic University, Kushtia 7003, Bangladesh; chayanali7@gmail.com; 3 Department of Pharmacy, BGC Trust University Bangladesh, Chittagong 4381, Bangladesh; largessvishal@gmail.com; 4 Department of Pathology, Dongguk University College of Medicine, Gyeongju 38066, Korea; taejung@dongguk.ac.kr

**Keywords:** NAGK, nsSNPs, polymorphism, molecular dynamics simulation, in silico

## Abstract

An enzyme of the mammalian amino-sugar metabolism pathway, *N*-acetylglucosamine kinase (NAGK), that synthesizes *N*-acetylglucosamine (GlcNAc)-6-phosphate, is reported to promote dynein functions during mitosis, axonal and dendritic growth, cell migration, and selective autophagy, which all are unrelated to its enzyme activity. As non-enzymatic structural functions can be altered by genetic variation, we made an effort in this study aimed at deciphering the pathological effect of nonsynonymous single-nucleotide polymorphisms (nsSNPs) in *NAGK* gene. An integrated computational approach, including molecular dynamics (MD) simulation and protein–protein docking simulation, was used to identify the damaging nsSNPs and their detailed structural and functional consequences. The analysis revealed the four most damaging variants (G11R, G32R, G120E, and A156D), which are highly conserved and functional, positioned in both small (G11R and G32R) and large (G120E and A156D) domains of NAGK. G11R is located in the ATP binding region, while variants present in the large domain (G120E and A156D) were found to induce substantial alterations in the structural organizations of both domains, including the ATP and substrate binding sites. Furthermore, all variants were found to reduce binding energy between NAGK and dynein subunit DYNLRB1, as revealed by protein–protein docking and MM-GBSA binding energy calculation supporting their deleteriousness on non-canonical function. We hope these findings will direct future studies to gain more insight into the role of these variants in the loss of NAGK function and their role in neurodevelopmental disorders.

## 1. Introduction

*N*-acetylglucosamine kinase (GlcNAc kinase or NAGK; E.C. 2.7.1.59) is a major enzyme from the sugar-kinase/Hsp70/actin superfamily, which is involved in the conversion of GlcNAc (*N*-acetylglucosamine) to GlcNAc-6-phosphate, a catalytic event present in amino sugar metabolism [1]. This metabolic pathway produces UDP-GlcNAc, which is the major substrate for the enzymes involved in protein *N*- and *O*-glycosylation and a substrate for sialic acid biosynthesis [2]. Since these post-translational modifications, *N*- and *O*-glycosylation, regulate a wide range of cellular processes, including various stress responses, transcription, and translation [3,4,5], NAGK plays a significant role in diverse cell signaling. Apart from that, our laboratory reported enzyme -independent functions, i.e., structural roles of NAGK, which was first identified when NAGK distribution was found differentially in different types of brain cells, including high expression in neurons and low expression in astrocytes and oligodendrocytes [6,7]. Structurally, NAGK is composed of 11 β-strands and 10 α-helices that are folded into an N-terminal small domain (1 to 117) and a C-terminal large domain (D^118^ to L^307^ and A^335^ to S^344^), arranged like a “V” shape. The active site of the enzyme is surrounded by the side of the β-sheet of a large domain with small domain helix α3 and divided as substrate and ATP binding sites. The primary substrate of NAGK, GlcNAc, binds in the substrate binding site, including small domain residues N^36^, S^76^ to D^79^, and D^107^, and also extends in the loop domain of T^127^ to N^130^ and G^145^ to D^152^. On the other hand, the conserved motifs in the small domain, including G^9^ to L^19^ of the PHOSPHATE1 motif, which forms β-turn, are involved in phosphate binding. The residues in the large domain, including V^269^ to L^275^, which are also known as the ADENOSINE motif, make interactions with the adenine base of the ATP [8,9,10].

It has been found that NAGK is involved in different stages of neuronal development, including dendritic arborization and axonal outgrowth, and this effect was unchanged even in the overexpressing kinase-deficient mutant NAGKs [11]. Mechanistic studies revealed the structural roles of NAGK, where the protein interacts with dynein light chain roadblock 1 (DYNLRB1) in dynein complex and promotes dynein functions in cellular growth [1], dendrites [6,7] and axon development [12], neuronal migration [13], and selective autophagy [14]. These observations suggest both enzymatic and non-enzymatic importance of NAGK in neuron and brain development. However, both of these functions can be altered by genetic variation, and therefore, in this study, we aimed to decipher the damaging effect of nonsynonymous single-nucleotide polymorphisms (nsSNPs) in the structural consequence of NAGK.

Single nucleotide polymorphisms (SNPs) are the most predominant types of genetic variation in humans, linked with various complex genetic and Mendelian disorders [12,13], and commonly occur in both coding and non-coding parts of a genomic region. The nsSNPs, which occurred in the coding region and caused amino acid substitutions, are the most responsible for phenotypic change and are associated with many genetic diseases due to their effect on protein structure, charge, solubility, stability, and function [15,16]. In light of this fact, it is expected that the presence of nsSNPs in NAGK might have a considerable impact on both canonical and non-canonical functions; however, analyzing the impact of a large number of nsSNPs using an experimental approach is more time consuming and expensive [17,18]. Therefore, we investigate the possible damaging effects of nsSNPs in NAGK structural dynamics using bioinformatics and molecular simulation approaches. Integration of bioinformatics tools in the large number nsSNP analysis is a cost-effective approach, and together with molecular dynamics simulation, it provides detailed insight on the structural consequence [19].

In this study, we identified four potential deleterious SNPs in the *NAGK* gene (G11R, G32R, G120E, and A156D), disrupting the structural and dynamic integrity of the protein structure. All of these variants are found to influence the structural organization of the catalytic site and reduced binding energy of NAGK and DYNLRB1, supporting their deleteriousness on both canonical and non-canonical functions of NAGK.

## 2. Results

### 2.1. Identification of Deleterious nsSNP

**Figure 1:**
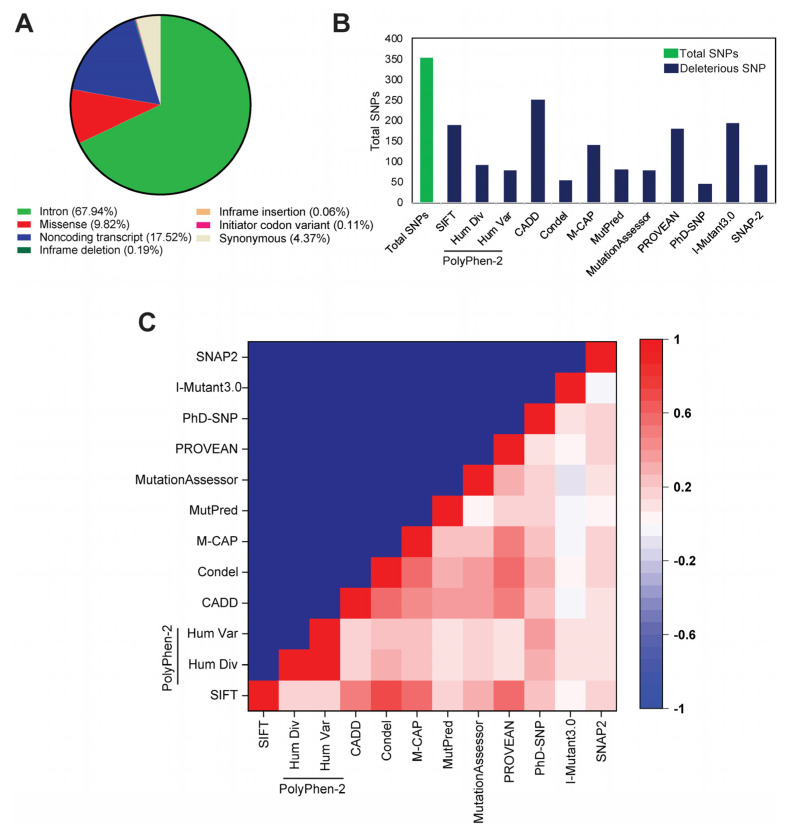
In silico-based identification of deleterious SNPs in the *NAGK* gene. (**A**) A pie chart highlighting the various types of SNPs in *NAGK*. (**B**) Bar plot describes the total number of missense SNPs and predicted deleterious SNPs by various algorithms. (**C**) Heatmap illustrating the pairwise correlation of predictions between various computational algorithms. The degree of correlation is highlighted by a color-coded map from red to white to blue; red indicates a positive correlation, white means neutral, and a negative correlation is denoted by blue color.

According to the dbSNP database, a total of 3596 SNPs were available for *NAGK.* Among those, 67.94% of SNPs were located in the intronic region, followed by 17.52% in the non-coding transcript, 9.82% missense, and 4.37% were synonymous with a 0.19% in-frame deletion, 0.11% initiator codon, and 0.06% in-frame insertion (Figure 1A). Concerning the vital role of missense variants on in vivo protein functions in various complex diseases [20,21], the present study only considered the missense SNPs to study their effects on NAGK structural dynamics.

A total of twelve deleterious predicting algorithms (Table S1, Supplementary File 2), which use either sequence or combined sequence and structure-based approach, were used to identify the most damaging ones from the retrieved 353 missense SNPs. Among these 12 used algorithms, the combined annotation-dependent depletion (CADD) algorithm recognized the highest number of deleterious SNPs (*n* = 251), whereas the lowest number of deleterious SNPs was identified by PhD-SNP (Figure 1B). Furthermore, the predictions of all algorithms were found to correlate significantly with each other independently, while I-mutant3.0 showed a slight negative correlation with MutationAssessor. Since each algorithm uses different parameters for SNP assessment, SNPs that retrieved more positive responses in different SNP algorithms are more likely to be deleterious [22,23]. Therefore, SNPs that were considered deleterious by at least ten different in silico algorithms were classified as high-risk nsSNPs in this study. The seven SNPs, rs762410705 (L68P), rs762422416 (G120E), rs773587630 (G11R), rs1235100397 (A156D), rs777835055 (A115D), rs1182635746 (G32R), and rs1190188472 (A160E), were identified as deleterious (Table S2, Supplementary File 2) by at least ten algorithms and these were considered as high-risk nsSNPs, and hence subjected to further analysis.

### 2.2. Conservation Analysis

Generally, residues, which are critical for protein stability, biomolecular interactions, and functions, are more usually conserved than others [22]. Therefore, SNPs in the conserved area are likely to be more pathogenic than those present in the variable region, disrupting structural stability, protein–protein interaction [24], and catalytic activity [25,26]. Since the NAGK non-canonical function mostly depends on diverse biomolecular interactions [1,6,7,12,13,14], we analyze the degree of amino acid conservation of NAGK to investigate further the possible impacts of high-risk SNPs based on evolutionary information. The ConSurf web server was used to predict the evolutionary conservation profile of NAGK, which is shown in Figure S1 (Supplementary File 2).

**Figure 2:**
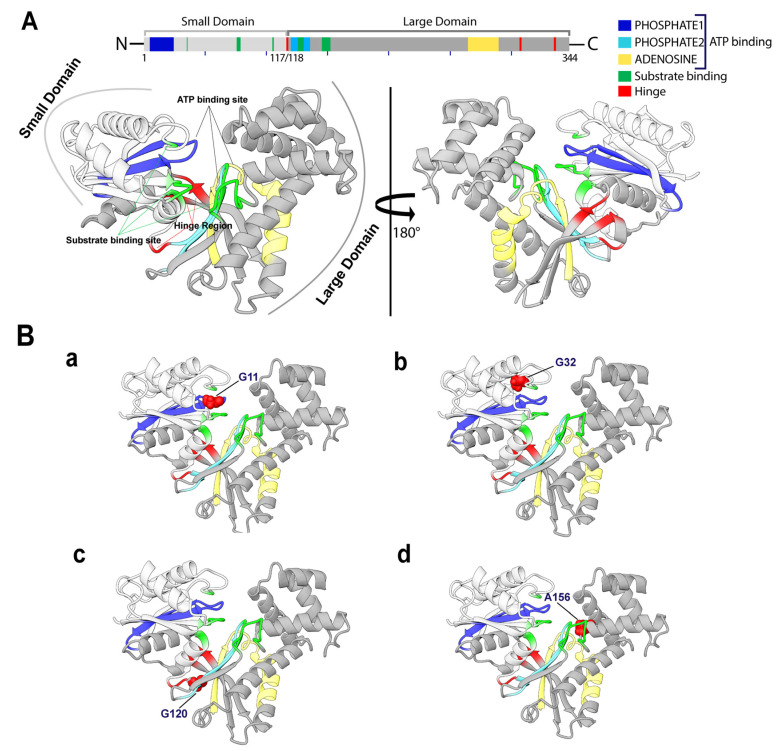
Molecular architecture in human NAGK crystal structure. (**A**) Representative three-dimensional structure highlighting the structural domains and motifs present in NAGK. (**B**) Cartoon representation labeled with identified variant position for G11R (**a**), G32R (**b**), G120E (**c**), and A156D (**d**).

According to the conservancy analysis, only four high-risk SNPs were found to be located in the highly conserved area: rs773587630 (G11R), rs1182635746 (G32R), rs762422416 (G120E), and rs1235100397 (A156D). Specifically, G^11^ and G^32^ residues are highly conserved and functional (Figure 2B(a,b)), while G^120^ and A^156^ residues are structural, highly conserved, and buried (Figure 2B(c,d)). Since these four SNPs (G11R, G32R, G120E, and A156D) are functionally conserved, they are likely to be more deleterious to the NAGK structure and function (Figure 2A), and hence their structural impacts are analyzed specifically through MD simulations.

### 2.3. Molecular Dynamics (MD) Simulation

To analyze the phenotypic consequence of the identified deleterious SNPs, NAGK conformational dynamics were visualized by conducting molecular dynamics simulation for 300 ns considering its wild and variant types. The simulation stabilities for all systems were characterized by RMSD analysis, representing that all systems achieved initial equilibrations after 75 ns and were maintained till the end. The wild-type showed a high deviation of RMSD (~4 Å) during the initial stage of the simulation (~50 ns); however, it retained stability afterwards, which remained until the end of the simulation (Figure S2A, Supplementary File 2). The G32R variant displayed RMSD pattern similar to the wild-type while showed slight deviations over the period (Figure S2C, Supplementary File 2). The rest of the mutants, G11R, G120E, and A156D, showed an increased RMSD deviation compared to the wild-type, and the fluctuation level reached near 4 Å in several timesteps in the total simulation (Figure S2B,D,E, Supplementary File 2). Since the last 125 ns of wild-type simulation (after 175 ns) showed a stable equilibration, the trajectories retained in this time length were considered for further analysis, which is also considered for all variants (Figure S2, Supplementary File 2).

The adequacy of the conformational sampling of the sub-trajectories (the last 125 ns trajectories) was confirmed by cosine content analysis of the first three principal components. The analysis indicates that the conformational samplings in the sub-trajectories are convergence, as all cosine content values of the principal components for this time window are lower than 0.7 (Table S3, Supplementary File 2) [27], where higher than indicated value represents insufficiency conformational sampling.

#### 2.3.1. Effects of Variants on Conformational Dynamics

**Figure 3:**
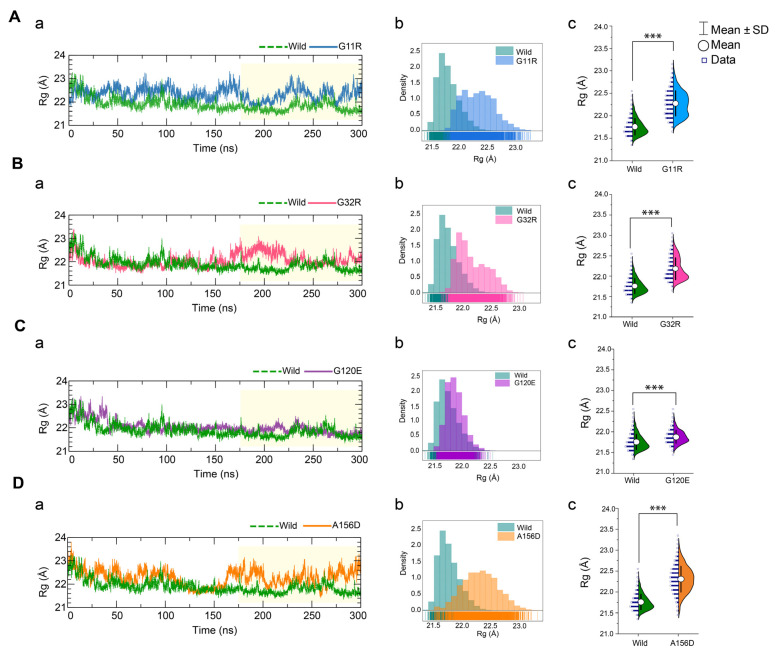
Analysis of radius of gyration deducing the changes in the NAGK conformational stabilities. Rg value of G11R (**A**), G32R (**B**), G120E (**C**), and A156D (**D**) variants versus wild-type NAGK, where in all cases, (**a**) represents time-dependent changes during the simulation, while (**b**) and (**c**) describe probability density and mean difference in the Rg distribution, based on the last 125 ns trajectories. The annotations used in **A**(**c**), **B**(**c**), **C**(**c**), and **D**(**c**) represent statistical significance, denoting *** *p* < 0.0001. Two-tailed, equal-sample variance Student’s *t*-tests were used to calculate the *p* values.

To analyze the NAGK conformational stability, the RMSD value of the sub-trajectories was again calculated as an indication of overall protein stability. The RMSD value, as compared to wild-type, was found higher in G120E and A156D than G11R (Figure S2F, Supplementary File 2). The RMSD values of G11R were also significantly higher than the wild-type, reflecting that all variants induced a higher structural deviation and thus flexibility. To further confirm this observation, radius gyration (Rg), another indicator of protein flexibility that indicates total protein compactness, was calculated and represented in Figure 3. The results also confirmed the substantial conformational changes in variant structures due to the high structural flexibilities. Compared to the wild-type, G11R showed a significant increase in Rg value during the last 125 ns of the simulation (Figure 3A(a)) and resulted in Rg distribution shifting right to the Rg of wild-type (Figure 3A(b)). Similar trends of Rg distribution were also observed in G32R and A156D mutants (Figure 3B,D), where A156D showed higher fluctuations in Rg than G32R during the last 125 ns. Instead, G120E showed a minor increase in the total Rg of the protein (Figure 3C), and the distribution was overlapped with the occurrence frequencies of wild-type, although the difference was statistically significant (Figure 3C(c)). Both RMSD and Rg analysis collectively suggest that all variants have higher structural flexibility than the native form, affecting overall protein stability.

**Figure 4:**
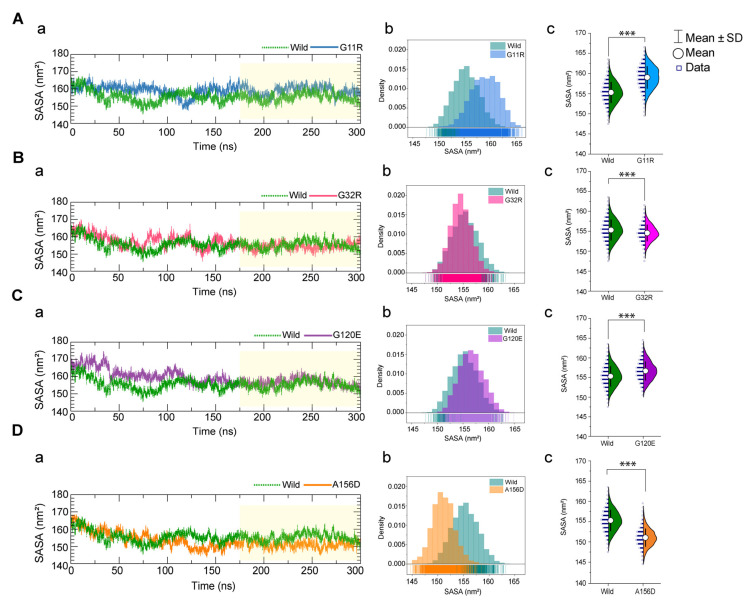
Analysis of total solvent accessible surface area (SASA) of the protein inferring the changes in the NAGK conformational stabilities. SASA value of G11R (**A**), G32R (**B**), G120E (**C**), and A156D (**D**) variants versus wild-type NAGK, where, in all cases, (**a**) represents time-dependent changes during the simulation, while (**b**) and (**c**) describe probability density and mean difference in the SASA distribution, based on the last 125 ns trajectories. The annotations used in **A**(**c**), **B**(**c**), **C**(**c**), and **D**(**c**) represent statistical significance, denoting *** *p*  <  0.0001. Two-tailed, equal-sample variance Student’s *t*-tests were used to calculate the *p* values.

Since the RMSD and Rg analysis indicated substantial structural changes in variant type structures, the total solvent accessible surface area (SASA) of all variants was calculated and compared with wild-type. The SASA indicates solvent accessibility, where a decrease in SASA describes the shrunken structure. At the same time, a high value denotes protein flexibility suggesting that the hydrophobic core of the protein appeared to be exposed in the aqueous surrounding due to the loss of hydrophobic interactions among nonpolar residue clusters [28]. As shown in Figure 4A,C, both G11R and G120E induced total SASA of the protein than the wild-type, and G11R showed higher deviation than the G120E. The SASA distribution of G11R shifted to the right than the wild-type and showed a high distribution between 160 to 163 nm^2^ (Figure 4A(b)). G120E represented high distribution in the range of 156 to 157 nm^2^, whereas the wild-type had a high distribution ranging from 156 to 158 nm^2^ (Figure 4C(b))_._ Conversely, A156D and G32R showed a reduced SASA value compared to the wild-type, in which A156D had a high distribution around 150 nm^2^ (Figure 4D(b)), while G32R raised SASA around 154 nm^2^ (Figure 4B(b)). The SASA analysis, together with RMSD and Rg calculations, thus summarizes that these variants caused the changes in overall protein dimension, which might lead to misfolded protein conformation and thus affect protein–protein interactions.

#### 2.3.2. Effects of Variants on Protein Dynamics

**Figure 5:**
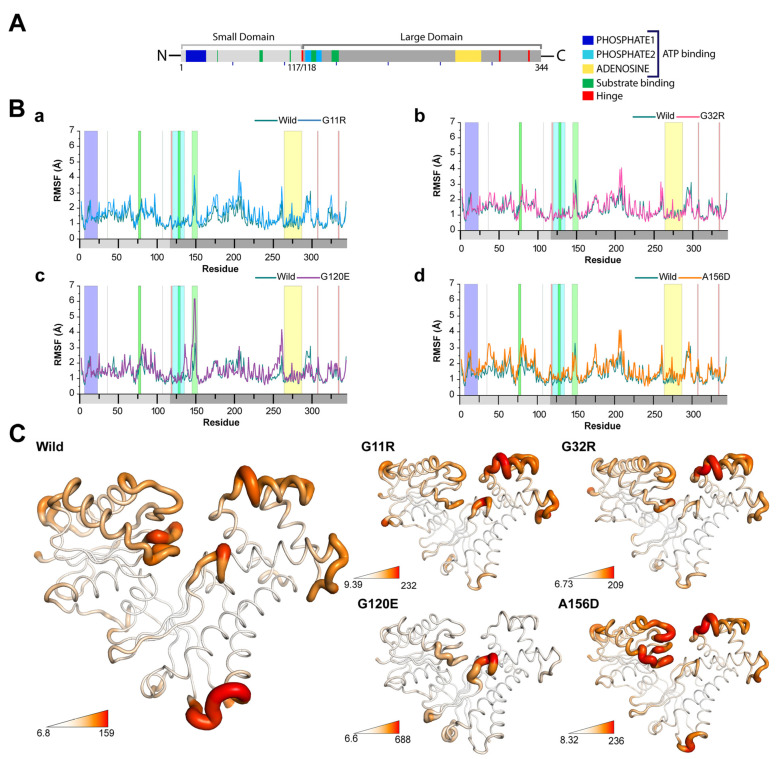
Alteration of residual flexibility in NAGK variants. (**A**) Map representing domains and motifs presents in NAGK involved in substrates and ATP binding. (**B**) The root mean square fluctuation (RMSF) plots highlighting the variations in structural flexibility between G11R (**a**), G32R (**b**), G120E (**c**), and A156D (**d**), compared to the wild-type. (**C**) The degree of flexibility is represented by the tube view of NAGK structure in various types with B-factor calculated from the RMSF analysis. The area having a high B-factor is shown as a broader tube with red shades, while a narrow tube with white shade means the regions have a low B-factor.

To recognize the effect of variants in the local flexibility of the protein, we calculated the root mean square fluctuations (RMSF) value of NAGK in both wild-type and variants, which indicates that amino acid substitution increased residual flexibility. As represented in RMSF analysis (Figure 5), it was revealed that all variants caused different fluctuations in the large domain-containing residues of the protein (including M^150^ to S^275^), where the residues in the NAGK small domain were seen to have a high fluctuation only in A156D (Figure 5B(d)). In the substrate binding site (Figure 5A), all variants changed the residual fluctuation in the loop domain, ranging from G^145^ to D^152^, where the degree of fluctuation was severe in G120E (Figure 5B(c)). G120E also induced high fluctuation in the residues of S^76^ to D^79^ of the substrate binding site, and this change was similarly found in A156D. In the case of G120E and A156D, the residue D^107^ was seen to fluctuate more than the wild-type, which was evidenced in the hydrogen bonding with GlcNAc (substrate of NAGK) and accepting proton during the nucleophilic attack on the γ-phosphate of ATP [11]. 

Similarly, N^36^ showed a high fluctuation in the case of A156D and other residues (H^37^ to I^40^), along with high fluctuations in G11R and G32R compared to the wild-type (Figure 5B(a,b)). The ATP binding site, containing conserved motifs such as ADENOSINE (residues, V^269^ to L^275^), was seen to have more fluctuation in A156D and G11R than the wild-type. In addition, residues in the region L^250^ to V^269^ and S^275^ to S^300^ showed reduced fluctuations in G120E and G32R. The residual fluctuations in the PHOSPHATE2 motif also deviated more in G120E and A156D; however, residual fluctuations in the hinge region remained unaffected. Additionally, G120E and A156D showed high RMSF values (Figure 5C,D) in the residues of D^79^ to R^85^ near the substrate binding site (S^76^ to D^79^).

**Figure 6:**
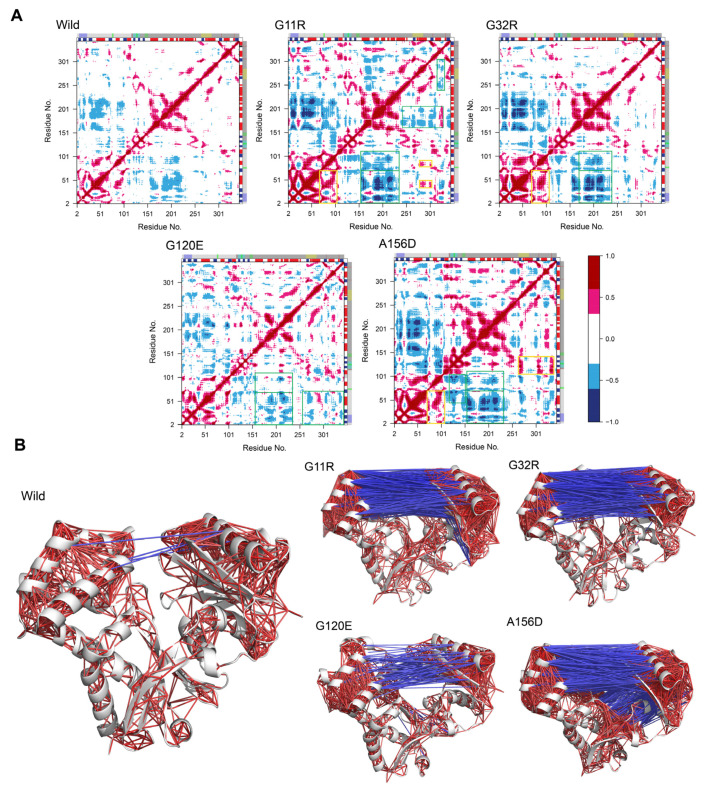
Effect of variants in the NAGK correlated motions. (**A**) Two-dimensional DCCM plot represents residual correlative motion by a color-coded heat map, where red denotes correlated motion between two residues, while blue indicates motion in an anti-correlated manner. Here, the color-coded bar from red to blue denotes the degree of correlation from 1 to −1. Either yellow or green boxes mark a specific area with positive and negative correlation in each map. On the other hand, the secondary structural elements (SSE) and domain organization are represented as the colored bars at the top and right of the map. The details of domain organization are discussed in Figure 5. Red and blue color in the SSE bar represents alpha-helix and beta-sheet, respectively. (**B**) Strongly correlative motion is represented by NAGK cartoon representation, where red lines indicate strongly correlated (6 to 8) motions, and blue are anti-correlated (−8 to −6) motions.

As RMSF highlighted the variants induced conformational changes in both substrate and ATP binding sites, we incorporated the dynamic cross-correlation map (DCCM) analysis to understand their effects on NAGK correlative motion. Figure 6A shows a color-coded DCCM for all systems, illustrating that variants induced a distinct change in the correlative motion of NAGK (Figure 6B). When the correlated motion is compared between wild-type and G11R, it is revealed that G11R increased both correlated and anti-correlated motions in NAGK. A substantial increase in the positive correlation was observed between the residues A^2^ to L^71^ and P^67^ to I^104^, while anti-correlated motion occurred between the residues E^240^ to T^303^ and F^302^ to D^330^. In addition, a positive correlation was found in the region between the region G^32^ to V^46^ and A^295^ to T^303^, and also between G^77^ to R^94^ and A^295^ to T^303^. Conversely, a considerable induction of anti-correlated motion was observed between the residues A^2^ to L^71^ and E^153^ to N^234^, and this region (E^153^ to G^243^) was also found to show a negative correlation with the residues of L^73^ to G^110^. Furthermore, G11R was also seen to induce anti-correlated motion between I^159^ to Y^205^ and L^242^ to H^325^. Similar trends in the anti-correlated motion were also found in G32R and A156D, specifically in the region between the residues A^2^ to L^71^ and E^153^ to G^243^ as well as the correlative motion between the region of residues A^2^ to L^71^ and P^67^ to I^104^. Unlike G32R and A156D, G120E showed reduced correlative and anti-correlated motions in these regions. Nevertheless, it showed mixed motion between the residues A^2^ to L^71^ and I^253^ to S^344^. A156D showed an increased anti-correlated motion in the region between the residues V^46^ to T^105^ and D^118^ to D^152^, and correlative motion between the region T^105^ to G^142^ and L^267^ to I^336^. The dynamic insight gained from DCCM analysis further supports that variants caused correlative motions in the substrate binding site and in the active site residues, specifically PHOSPHATE and ADENOSINE motif.

Again, to expose the changes in protein dynamics, we conducted principal component analysis (PCA), representing most of the dominant motions during the simulations [17,29]. Remarkably, all variants were found to have more variances than that of the wild-type, such as G11R, G32R, G120E, and A156D with variances of 60.9%, 53.9%, 53.9%, and 58.8%, respectively, in the first three PCs, whereas wild-type has a variance of 43.2%. All variants also produced different patterns in the directional movement of NAGK, as highlighted by root mean square inner product (RMSIP) analysis (Figure S3, Supplementary File 2), that predict similarities between the subspaces of wild-type and variants trajectories by comparing covariance matrices. A perfect similarity is indicated by RMSIP value 1, while 0 means the similarities between the matrices are orthogonal. When compared to the wild-type, all variants showed a minimal RMSIP value, lower than 0.5, which suggests that structural dynamics between the wild-type and variants are hardly identical, meaning that variants induced a loss of cumulative movements [30,31].

To compare the provable conjoined movements, eigenvectors (EV) of the first three PCs were drawn in a two-dimensional plot, indicating conformational distribution states by color-coded dot representation, described briefly in Figure S4. As shown in Figure S4A (Supplementary File 2), the conformational distribution in the wild-type on the projection of PC 1/3 and 2/3 was more distinct, which indicates a substantial energy barrier [32]. However, all variants expect G120E showed overlap in PC subspaces, suggesting a loss of periodic conformational shifting due to the lack of an energy barrier. However, G120E showed high conformational distribution in intermediate states and produced energetically fewer stable states (scattered blue region) than the wild-type (Figure S4D, Supplementary File 2). This observation confirmed that G120E increased fluctuations in NAGK domains and suggested a loss of coordinated motions [33].

**Figure 7:**
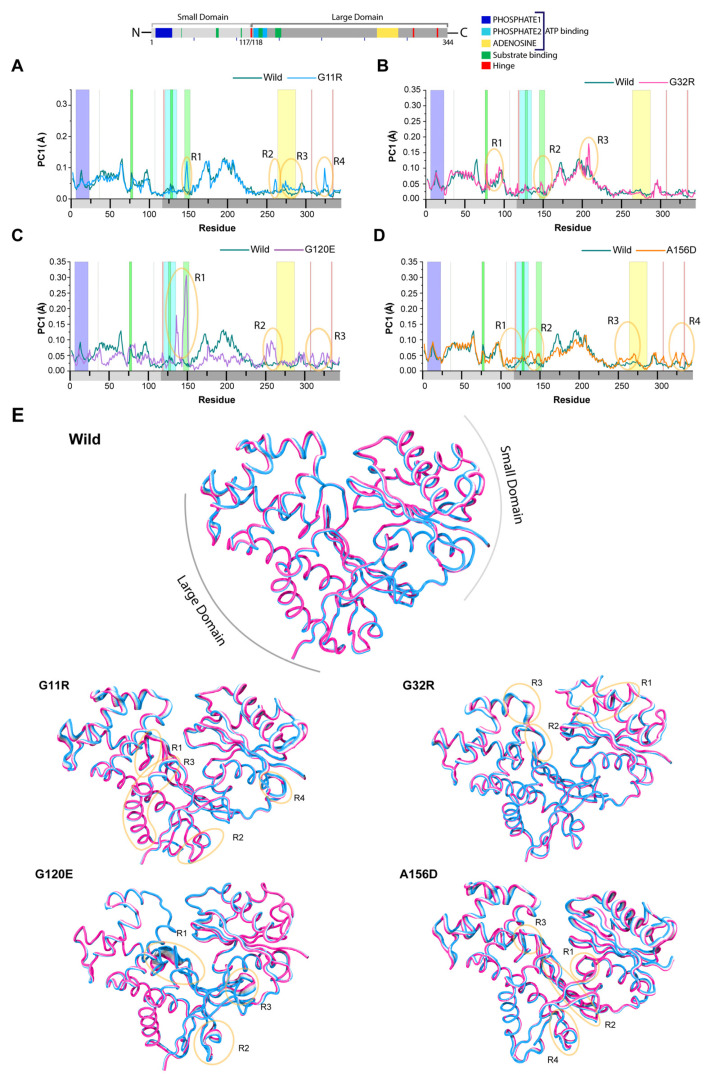
Variants induced changes in NAGK dynamical motion. Line plot showing the deviation between the first principal component of NAGK in variants (G11R (**A**), G32R (**B**), G120E (**C**), and A156D (**D**)) and wild-type. (**E**) The alterations in atomic movements in PC1 are demonstrated by the tube model, where widen tube indicates an area with high flexibility marked by an orange circle.

The residual mobility in NAGK variants, which was highlighted by PC1, is visualized in Figure 7 and compared with wild-type. PC1 plot showed that variants including G11R, G32R, G120E, and A156D induced high residual mobility in the substrate binding site including the region G^145^ to D^152^, where high induction was observed in G120E (Figure 7C). G11R showed reduced mobility in the region of substrate binding site (residues S^76^ to D^79^), but induced mobility in the ADENOSINE binding motif of the ATP binding site (Figure 7A,E). On the other hand, G120E showed reduced mobility in the PHOSPHATE1 motif, while increased residual mobility in the hinge regions of residues D^118^ to G^119^ and L^307^ to R^308^ and beside the adenosine binding motif (residues, L^250^ to V^269^). A156D also produced high mobility in all hinge regions as well as in PHOSPHATE2 and adenosine motifs in the ATP binding regions (Figure 7D). Both variants, G120E and A156D, reduced flexibilities in the region of residues, M^188^ to R^206^, where G120E also reduced residual mobility in T^35^ to N^50^ as well as changed the degree of mobility in G^78^ to F^95^. However, A156D in this region (D^79^ to L^87^) reduced residual mobility.

#### 2.3.3. Variants Alters NAGK Secondary Structural Organization

To measure the effect of variants in the NAGK secondary structural organization, we used the Define Secondary Structure of Proteins (DSSP) algorithm on wild-type and variant simulation trajectories to analyze the changes in NAGK secondary structure elements (Figures S5 and S6, Supplementary File 2). As shown in Figure S5B, G11R induced more bend and B-sheet conformation in the PHOSPHATE1 loop than the wild-type (Figure S5A). In addition, G11R introduced more turn conformation in the residues H^148^ to S^155^, where at this region, wild-type showed more bending conformation. Furthermore, G11R introduced more bend conformation in the residues F^208^ to R^212^ and decreased A-helix conformation. In the C-terminal region (D^330^ to S^340^), G11R produced more bend and coil conformation and reduced A-helix formation during the simulation. A similar trend of the A-helix formation was also revealed in the C-terminal region (D^330^ to S^340^) of the G32R variant (Figure S5C), and G32R also introduced more B-sheet and bend conformation in the phosphate1 loop. In addition, G32R caused more turn conformation in the residues of L^75^ to I^87^ instead of coil formation. In contrast, G120E altered conformational stability more than the wild-type, reducing the proportion of helix and B-sheet occupancies. G120E showed a substantial reduction in the B-sheet formation in residues G^120^ to A^160^, while induced more turn conformation. Moreover, in the region L^75^ to E^100^, G120E increased the formation 3-helix and turn conformation by disrupting A-helix formation (Figure S5D). In the C-terminal region (L^250^ to G^270^), G120E reduced 3-helix formation; instead, it introduced turn conformation. A156D reduced A-helix formation in the NAGK large domain (Figure S5E), especially in the region of residues E^240^ to S^344^. Like G120E, A156D also reduced 3-helix formation in residues L^250^ to G^270^, and also A-helix in D^330^ to S^340^ while inducing 3-helix formation in this region. An induction of turn and 3-helix formations were also observed in the residues H^148^ to S^155^ in the case of A156D (Figure S6).

### 2.4. Impacts on Non-Canonical Functions

**Figure 8:**
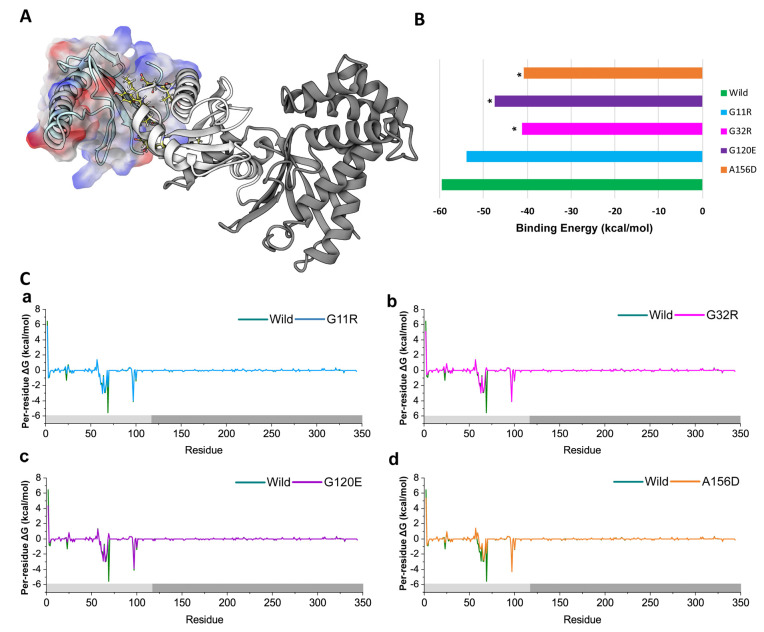
Molecular insights into the change of protein-protein binding of NAGK induced by variants. (**A**) Protein-protein docking highlighting the intermolecular interaction between NAGK-DYNLRB1 complex. (**B**) MM-GBSA binding energy profile describing the change of binding affinity in NAGK wild/variants-DYNLRB1 complex. Here, the star indicates more than 10% variation in energy compared to wild-type. (**C**) Per-residue decomposition analysis shows the binding contribution of NAGK in NAGK-DYNLRB1 complex for G11R (**a**), G32R (**b**), G120E (**c**), and A156D (**d**), compared to wild-type.

Our previous report evidenced the physical interaction of NAGK in the dynein complex, where it interacts with DYNLRB1 by its small domain and promotes dynein-mediated functions [11,13,14,34]. To analyze the effect of identified NAGK variants in dynein association (Figure 8A), we analyze the binding energy between NAGK and DYNLRB1in both wild-type and variants (Figure 8B). Resultantly, all variants showed a reduction in the binding energy of the NAGK-DYNLRB1 complex as a result of the amino acid change (Table S4, Supplementary File 2). The binding energy variation between wild-type and variants was calculated, which represents that binding energy between these two proteins is reduced to >8% in all variants (Figure 8B). Among all the variants, A156D and G32R highly decreased the binding energy to >30%, having a binding energy of −40.86 kcal/mol and −41.21kcal/mol, respectively, whereas the wild-type showed binding energy of −59.45 kcal/mol. On the other hand, G11R and G120E also showed less binding energy than the wild-type, which is −53.97 kcal/mol and −47.36 kcal/mol, respectively. Figure 8C represents the deleterious effect of variants in protein–protein interaction, which showed that variants reduced the total binding free energy contributed by hot-spots, especially K^59^AGVDPLVPLR^69^ [13,14]. In wild-type, the R69 residue showed total contributing binding energy of −5.58 kcal/mol, which, however, reduced in G11R, G32R, G120E, and A156D to −2.17, 0.34, 0.72, and −1.86 kcal/mol, respectively (Figure 8C).

## 3. Discussion

In this study, a series of analyses have been incorporated to understand the changes in NAGK structural and conformational dynamics caused by missense variants identified by various bioinformatics tools. Previous studies on NAGK crystal structure suggested that NAGK confers large conformational changes during the catalytic process, like other proteins from kinase/Hsp70/actin superfamily, allowing the small and large domains to be closely connected with substrates. However, this dynamic change is not only critical for substrates selectivity but also for its structural functions, which encouraged us to examine the dynamic nature of NAGK in the presence of deleterious variants.

To identify the potential deleterious missense SNPs, we used different bioinformatics tools with various features and parameters to minimize the prediction errors and maximize the reliability and accuracy of the prediction [15,35]. Thus, by incorporating 11 bioinformatics tools, consisting of 12 algorithms, we identified seven high-risk SNPs among the 353 missense SNPs, of which were agreed by at least 10 algorithms and thus can be considered reliable for further analysis [15,16].

Accumulating studies in functional genomics evidenced the fundamental roles of evolutionary information in detecting the disease-causing mutations [36,37]. Thus, for rational prioritizing the identified high-risk SNPs, we considered the evolutionary conservation of NAGK, which was performed in the ConSurf server. As highlighted in Figure S1, four potential variants, G11R, G32R, G120E, and A156D, are highly damaging because their positions are located in highly conserved areas. Interestingly, G11R is located in the PHOSPHATE1 motif of the ATP binding site of the small domain, and it was illustrated earlier that G11 makes direct interaction with the ATP-Mg^2+^ and coordinates nucleophilic attack of the γ–phosphate group [38]. Thus, the replacement of neutral residue (Glycine) with highly positive charged residue (Arginine) in G11R would certainly disrupt binding positively charged atoms in ATP.

On the other hand, various analyses from MD simulation, such as RMSD and Rg analysis, revealed the structural changes in NAGK in both wild-type and variant form, concluding that variants induced a substantial increase in structural flexibility. In support of this finding, SASA analysis added that the high flexibility induced by all variants, except G120E, may lead to expanding solvent-exposed area, resulting in protein misfolding and being deemed responsible for loss of function. A decreased SASA value, which was observed for G120E, suggested decreasing total protein solubility, which can also be a negative factor of protein–protein interactions [39].

All of the variants also showed different correlative motions compared to wild-type NAGK. The correlation analyses show a high anti-correlation between the domains induced in the variants, indicating global conformational changes so that the domains of the NAGK are pushed away from each other. Furthermore, the conformational ensembles of wild-type structure in PCA analysis were clustered into two distinct metastable states divided by a substantial energy barrier. These distinct clusters might provide an essential aspect of the control mechanism and suggest that NAGK performs periodic conformational shifting to reorient its terminal domains. As previous study evidenced, substrate-binding in NAGK differs depending on domain movement, from open to close formation, where the closed configuration ensures the tight binding of substrates [8]. Hence, DCCM and PCA collectively evidenced that variants changed essential correlated and coordinated movements, which eventually leads to loss of function.

The loss of structural stability and function is correlated with increased motion, which is either in the active or functional interacting site, and caused substantial changes in the secondary structural organization in such a way that it impairs the potential ligands bindings [40]. The results from RMSF, DCCM, PCA, and DSSP analyses provide evidence for disrupted structural activity in all variants, particularly around the active sites, which is thus believed to be associated with the loss of ligand bindings. In G11R, the variants showed substantial structural changes near the active site and induced high correlative motions, indicating the influence of G11R on the NAGK structural function, which is also supported by MM-GBSA analysis. G32R, on the other hand, is also located in the small domain and was demonstrated to induce flexibility in the substrate binding site (Figure 5A). Indeed, G32R caused substantial 3-helix formation instead of loop conformation of substrate binding site, specifically in G^145^ to D^152^ (Figure S6C, Supplementary File 2). This indicates that G32R disrupts the plasticity of the substrate binding site, which might affect the substrate binding and substrate selectivity. In addition, G32R was also shown to induce both correlative and random motion (Figure 6B) and reduced substantial binding energy in protein–protein interaction (Figure 8B), indicating a damaging impact on NAGK structurally related functions.

Variants in the large domain, G120E and A156D, albeit not present in the small domain, modulate structural integrity of both small and large domains, where G120E reduced both B-sheet and helix formation in most of the region of the NAGK structure (Figure S5). G120E markedly reduced A-helix formation in the PHOSPHATE2 and ADENOSINE motifs, indicating that the damaging effect of G120E in the ATP binding (Figure S6D, Supplementary File 2). Furthermore, G120E induced higher flexibility in the loop region of the substrate binding site and reduced the correlative domain motion, suggesting that G120E may affect NAGK substrate selectivity. A156D induced more correlative motions than the other variants and induced changes in the helix conformation in the residues E^240^ to S^344^ containing the ADENOSINE motif (Figure S6E, Supplementary File 2). This observation further indicates the damaging roles of A156D in both canonical and non-canonical functions of NAGK.

## 4. Materials and Methods

### 4.1. Data Collection and Identification of Deleterious SNPs

The National Center for Biological Information (NCBI) SNP database (dbSNP) provides detailed information about the single nucleotide variations of any gene sequence, and this database was used for collecting relevant *NAGK* gene variations along with their respective rs IDs. A total of eleven in silico damaging SNP prediction tools were used for the identification of the most damaging SNP prediction, which are sorting intolerant from tolerant (SIFT), polymorphism phenotyping v2 (Polyphen-2), Combined Annotation-Dependent Depletion (CADD), Consensus deleteriousness score of missense mutations (Condel), M-CAP, MutPred, MutationAssessor, protein variation effect analyzer (PROVEAN), predictor of human deleterious single nucleotide polymorphisms (Phd-SNP), I-Mutant3.0, and SNAP2. Here, the prediction method by the SIFT tool is based on the PSI-Blast algorithm [41], which includes sequence homology and physical properties of amino acids [42] for determining the tolerated or desecrated substitution in every site of the sequence [43]. PolyPhen-2 uses probabilistic classifier to analyze the functional significance of an allele change and the mutation effect by Naïve Bayes algorithm [44,45]. PolyPhen-2 prediction also depends on the number of sequences, phylogenetic, and structural properties characterizing the substitution [46]. In the present study, two models of polyphen-2 were used, HumDiv and HumVar, where HumVar identifies extreme phenotypes while HumDiv classifies less damaging SNPs using position-specific independent counts [47]. CADD is an integrative annotation tool that can score human single nucleotide variants and short insertions and deletions based on more than 60 genomic features [48] and can effectively prioritize causal variants in genetic analyses, particularly highly penetrant contributors to severe Mendelian disorders [48]. Condel evaluates the probability of missense single nucleotide variants (SNVs) deleterious. It computes a weighted average of the scores of SIFT, PolyPhen-2, MutationAssessor, and FatHMM [49,50]. M-Cap is a clinical pathogenicity classifying tool that correctly dismisses 60% of rare missense variants of uncertain significance in a typical genome at 95% sensitivity [51]. The MutPred tool using random forest classifier can categorize an amino acid substitution as either deleterious/disease-associated or neutral, based on three classes of attributes, the evolutionary conservation of the protein sequence, the protein structure and dynamics, and can determine the changes in atomic and molecular level induced by the amino acid substitution [52,53]. Prediction of the functional effect of a mutation was investigated by MutationAssessor [54], depending on sequence conservation, using multiple sequence alignments [53,55]. PROVEAN [56] is a sequence-based prediction tool that estimates the effect of protein sequence variation on protein function [42,57]. The effect of damaging nsSNPs was determined in the protein sequence by applying delta alignment scores based on variant version and reference of the protein sequence [57]. Phd-SNP [58] software was also used to investigate the effect of mutation on protein function [59]. From the neutral protein, it segregate SNPs related to Mendelian and complex diseases by using evolutionary information [58]. I-mutant3.0 predictor uses Support Vector Machine (SVM) algorithm, which can estimate the stability change, donated by ΔΔG value (kcal/mol), upon single-site mutation based on a protein structure or sequence [60]. The DDG (kcal/mol) value and RI value (reliability index) of mutant are calculated by I-mutant3.0. SNAP2 is based on a learning device method known as neural network, utilizing the information of automatically created multiple sequence alignment and some structural features for prediction of mutation impact on protein function [61,62].

### 4.2. Conservation Analysis

To perform the conservation analysis of the native *NAGK* gene, the Consurf web tool was used [26]. This web tool analyses the evolutionary pattern of amino acid or nucleic acids (DNA/RNA) of the macromolecule substitutions among homologous sequences to reveal regions that are important for structure and/or function [63,64]. The Bayesian calculation method was used to calculate the conservation scores from the protein sequence. We considered those nsSNPs of *NAGK* that were found in the highly conserved region for further analyses. A score from 1 to 4 was considered as a variable, whereas scores between 5 and 6, and 7 to 9 were considered as intermediate and conserved, respectively.

### 4.3. Molecular Dynamics (MD) Simulation

#### 4.3.1. Preparation of Simulation System

To perform molecular dynamics simulation, the three-dimensional crystal structure of NAGK was first retrieved from the protein data bank (PDB ID: 2CH6) and prepared the following energy minimization procedure with Optimized Potential for Liquid Simulations (version 3e), as described earlier [13,14,16,65,66,67]. After preparing the structure, Schrödinger 2017-1 (Schrödinger, LLC, New York, NY, USA, 2017) was used to include respective variants (G11R, G32R, G120E, and A156D) in the structure by using mutant residue script. We further energy minimized the structures (wild-type and variants) using MD refine script of YASARA Dynamics software (YASARA Biosciences GmBH, Vienna, Austria), which follows a short MD simulation for 0.5 ns using the YAMBER3 force field [68]. Other aspects of this short simulation were described earlier [15,65,69]. The lowest energy conformer from the MD ensemble was used for further analysis.

MD simulation was performed by YASARA Dynamics software by using Assisted Model Building with Energy Refinement (AMBER 14) force field [70,71], as previously described [17,72,73,74]. In the beginning, structure, variants, and native were cleaned and the hydrogen bond network was optimized. A cubic simulation cell was generated, which was 10 Å more extended than the protein on each side. The transferable intermolecular potential3 points (TIP3P) water model was used for solvating the system [75]. The protonation state of each amino acid was maintained correctly with a combination of the H-bonding network optimization and SCWRL algorithm [76]. The acid dissociation constant value (pKa) for amino acids was also calculated using Ewald summation [77]. NaCl ions were added with a physiological concentration of 0.9% (0.15 M NaCl), with additional counter ions (Na^+^ or Cl^-^) to neutralize the cell. Following the simulated annealing method, the system was energy minimized using the steepest gradient approach (5000 cycles). At physiological conditions of (T = 298 K, pH = 7.4, 0.9% NaCl) [78], MD simulation was performed for 300 ns, using particle-mesh Ewald (PME) method to explain the long-range electrostatic interactions at a cut off distance of 8 Å [79]. During the simulation, constant pressure and Berendsen thermostat were maintained, and a multiple timestep algorithm was used to set a time step interval of 2.00 fs [80,81]. Each simulation trajectory with 50 ps time interval was acquired and analyzed by various evaluative measures viz. RMSD, RMSF, Rg, and SASA of protein backbone using default script of YASARA [82] and VMD software (Version 1.9.3, 2016, Theoretical and Computational Biophysics Group, Urbana, IL, USA) [83,84,85]. The secondary structure elements of all simulated trajectories were calculated by DSSP software (EMBL, Heidelberg, Germany) [86,87]. In addition, DCCM and PCA analyses were performed by the Bio3D [88] package integrated with R program. The DCCM analysis is a popular method for analyzing the trajectories to explore the inner protein dynamics [89], providing detailed insight on the correlative motion of protein. On the other hand, PCA shows the dominant collective motions of biological systems by reducing the dimensionality of large ensembles [66]. The mathematical of DCCM and PCA have been described previously [15,16,65,69].

The first 3 principal components were used to calculate the cosine content, which indicates the statistical significance of convergence of the trajectories, which was accomplished using the essential dynamics program of GROMACS simulation package. Here, a cosine value lower than 0.7 is considered a good convergence, while values close to 1 indicate that the simulation is not converged [90]. To analyze the similarity between two sets of modes obtained from different principal components (wild-type or variant), the root-mean-square inner product (RMSIP) was calculated by using Bio3D [91]. The root-mean-square inner product (RMSIP) over the first ten eigenvectors of the Cα atoms, the value varies from 0 to1, where 0 means the similarity is orthogonal while 1 indicates identical directionality [27,92].

#### 4.3.2. Protein–Protein Docking and MM-GBSA Calculation

To predict the interacting model of NAGK–DYNLRB1 (PDB ID: 2HZ5, Chain B), protein–protein docking was performed in the SwarmDock server [93], which preserves protein flexibility during the docking calculation [94]. After that, mutant residue script from Schrödinger 2017-1 software was used again to introduce respective variants (G11R, G32R, G120E, A156D) in the NAGK structure of the docked complex. To refine the mutant NAGK_wild/variant_-DYNLRB1 complex, a short MD simulation (0.5 ns) was again performed using the similar approach described earlier. The lowest energy conformer from MD refine was used for binding energy calculation by MM-GBSA method using the following equation:

$$∆G_{bind}=G_{com}-\left(G_{Protein\_rec}+G_{Protein\_lig}\right)$$

$$\;\;\;\;\;\;\;\;∆G_{bind}=∆H-T∆S\approx E_{MM}+∆G_{sol}-T∆S\;$$

Here, the change in total free energy between bound-state (*G_com_*) and unbound-state systems (*G_protein_*__*rec*_ + *G_protein_*__*lig*_) is defined by ∆*G_bind_*_._ ∆*G_bind_* can be described into three additional terms: ∆E_MM_ represents total gas-phase energy, which is the total of ∆*E_internal_*, ∆*E_electrostatic_*, and ∆*E_vdw_*); ∆*G_sol_* describes the total energy of polar (∆*G_GB_*) and nonpolar (∆*G_SA_*) contributions to solvation; and the conformational entropy upon the binding is described by T∆S.

Here, the calculation was performed by assigning Amber ff02 force field to the protein. Here, Onufriev et al.’s GB^OBC1^ model [95] was used to measure the desolvation of polar elements, where the dielectric constants were considered 80 for the solvent and 1 for the solute. The LCPO algorithm [96] was used for the nonpolar elements desolvation, considering the values 0.0072 and 0 for γ and b, respectively. In the calculation, entropies were not considered due to low prediction accuracy [97]. All of the calculation was performed in the HawkDock web server [98] and the complex preparation method is described in detail elsewhere [99].

### 4.4. Statistical Analysis

In this study, correlation among different bioinformatics tools was predicted by SPSS v19 software (IBM, Armonk, NY, USA). To find out the most significant combinations, *t*-tests and single-factor ANOVA tests were applied. GraphPad Prism v 8.0 (GraphPad Software, San Diego, CA, USA) software was used to analyze the MD trajectories statistically. To compare the simulation results, *p* values of < 0.0001 were considered to be highly significant, which were performed with two-tailed, equal-sample variance Student’s *t*-tests.

## 5. Conclusions

In summary, the identified NAGK missense variants, G11R, G32R, G120E, and A156D, are potentially damaging to NAGK structure and function. All of these variants were seen to disrupt the integrity of the catalytic site, induce high correlative motions, and reduce the binding energy of NAGK to DYNLRB1, indicating their damaging effect on NAGK general structural functions. Although further experimental verifications to assess the effect of these variants in NAGK functions is recommended, this study provides a starting point for further investigation of their associations in various brain developmental genetic disorders. The knowledge gained from this study may be directly helpful in efforts toward gene-based therapy and CRISPRs.

## Data Availability

The data presented in this study are available on request from the corresponding author.

## Supplementary Materials

The following are available online at https://www.mdpi.com/article/10.3390/ijms22158048/s1.
