# Supplementary material for: Computational Insights into the Deleterious Impacts of Missense Variants on N-Acetyl-d-glucosamine Kinase Structure and Function
Source: Int J Mol Sci. 2021 Jul 28;22(15):8048. doi: 10.3390/ijms22158048 (PMC8347710; doi:10.3390/ijms22158048)
Supplement: Supplementary file 1 [file ijms-22-08048-s001.zip › ijms-1243023-supplementary/Supplimentary File/Revised Supplimentary File 2.pdf]

Supplementary File 2

Table S1: The prediction cut-off used for the identification of deleterious SNPs of *NAGK*

Table S2. Cumulative prediction of damaging nsSNPs in *NAGK*

Table S3. The cosine content of the first three principal components was calculated for this sub-trajectory (the last 125 ns of MD simulation).

Table S4. Change of binding free energy (kcal/mol) of NAGK-DYNLRB1 revealed by MM-GBSA binding energy calculation.

Figure S1. Analysis of Consurf web server representing the conservation profile of amino acids in NAGK.

Figure S2. Evaluation of simulation stabilities. (A) Comparative root-mean-square deviations (RMSDs) analysis of Cα atoms for wild-type and NAGK variants, where green line demonstrates the wild-type, and the blue, pink, violet, and orange lines represent G11R, D32R, G120E, and A156D, respectively. RMSD plot of G11R (B), G32R (C), G120E (D), and A156D (E) variants as compared with NAGK wild-type. The last 125 ns of trajectories of systems, as highlighted by the yellow shade, were considered for further analysis. (F) Half violin plot describes probability density and mean difference in the RMSD distribution, based on the last 125 ns trajectories. The blue, pink, violet, and orange lines indicate G11R, G32R, G120E, and A156D, respectively, while green denotes wild-type. The annotations represent statistically significant, denoting *** $p < 0.0001$. Two-tailed, equal-sample variance Student's t-tests were used to calculate the P values.

Figure S3. The root mean square inner product (RMSIP) value was analyzed by overlapping matrix for the first ten principal components from sub-trajectories of wild and variants, including G11R (a), G32R (b), G120E (c), and A156D (d). The grey gradient visualizes the RMSIP values from white to black.

Figure S4. Principle Component Analysis (PCA) of NAGK dynamics in different systems, including wild (A), G11R (B), G32R (C), G120E (D), and A156D (E). The conformational distribution of each protein in a different system was captured on by the first three principal components, where a single protein conformer is represented by each dot with the simulation time, represented by a color-coded scale from blue to white to red. The variance captured by eigenvectors is also represented.

Figure S5. Variants induced changes in the secondary structure elements of NAGK. (A) Wild-type, (B) G11R, (C) G32R, (D) G120E and (E) A156D.

Figure S6. Residue-wise average secondary structural occupancies for wild (A), G11R (B), G32R (C), G120E (D), and A156D (E) structure, during the last 125 ns simulation. Here, dark blue color stands for Beta-sheet and red color for alpha-helix conformation.

Table S1. The prediction cut-off used for the identification of deleterious SNPs of *NAGK*

Name of tool		Prediction method	Cut-off value
SIFT		Sequence	≤0.05
Polyphen-2	HumDiv	Sequence and structure	>0.9
	HumVar	Sequence and structure	>0.9
PROVEAN		Sequence	≤−2.5
CADD		Sequence	>20
Condel		Sequence	>0.9
M-CAP		Sequence	>0.025
MutPred		Sequence and structure	>0.75
MutationAssessor		Sequence and structure	>2
PhD-SNP		Sequence	>0.5
I-Mutant 3.0		Sequence and structure	<−0.5
SNAP2		Sequence and structure	>50

Table S2. Cumulative prediction of damaging nsSNPs in *NAGK*

RS ID	Substitution	SIFT	CADD	Condel	M-CAP	MutPred	MutationAssessor	PROVEAN	PhD-SNP	Polyphen-2 HumDiv	Polyphen-2 HumVar	Imutant3.0	SNAP-2
rs762410705[a]	L68P	**0**	**31**	**0.945**	**0.066**	**0.765**	**3.08**	**-6.53**	**0.569**	**1**	**1**	**-0.68**	**73**
rs762422416[a]	G120E	**0**	**32**	**0.945**	**0.129**	**0.854**	**3.195**	**-7.63**	**0.59**	**1**	**1**	**-0.91**	**81**
rs773587630	G11R	**0**	**34**	**0.945**	**0.344**	**0.874**	**3.195**	**-6.36**	**0.863**	**1**	**1**	-0.01	**92**
rs777835055	A115D	**0**	**29.3**	0.895	**0.050**	**0.845**	**2.91**	**-4.27**	**0.659**	**1**	**0.995**	**-1.06**	-51
rs1182635746	G32R	**0**	**32**	**0.935**	**0.058**	0.739	**3.15**	**-7.04**	**0.639**	**1**	**0.999**	-0.24	**51**
rs1190188472	A160E	**0**	**26.5**	0.697	**0.049**	**0.789**	.	**-3.56**	**0.773**	**0.998**	**0.979**	**-0.51**	**59**
rs1235100397	A156D	**0**	**28.2**	**0.906**	**0.067**	**0.813**	.	**-5.63**	**0.839**	**1**	**0.994**	**-1.77**	**64**

The bold value in each tool indicate the respective SNP is deleterious, which satisfied the following criterion, SIFT (≤0.05), polyphen-2 HumDiv (>0.9), Polyphen-2 HumVar (>0.9), PROVEAN (≤−2.5), CADD (>20), Condel (>0.9), M-CAP (>0.025), MutPred (>0.75), MutationAssessor (>2), PhD-SNP (>0.5), I-Mutant3.0 (<−0.5), and SNAP-2 (>50). [a]Highly pathogenic SNP, which was agreed to by all predictors.

Table S3. The cosine content of the first three principal components was calculated for this sub-trajectory (last 125 ns).

System Name	PC1	PC2	PC3
Wild	0.641	0.241	0.149
G11R	0.407	0.055	0.202
G32R	0.0004	0.011	0.058
G120E	0.329	0.015	0.070
A156D	0.6007	0.245	0.026

Table S4. Change of binding free energy (kcal/mol) of NAGK-DYNLRB1 revealed by MM-GBSA binding energy calculation.

System	ΔE_{VDW}	ΔE_{ELE}	ΔG_{GB}	ΔG_{SA}	ΔG_{bind}
Wild	-73.44	-387.81	413.02	-11.22	-59.45
G11R	-72.92	-277.43	307.06	-10.68	-53.97
G32R	-70.15	-196.37	234.84	-9.53	-41.21
G120E	-71.23	-308.26	342.28	-10.15	-47.36
A156D	-71.95	-266.82	307.74	-9.83	-40.86

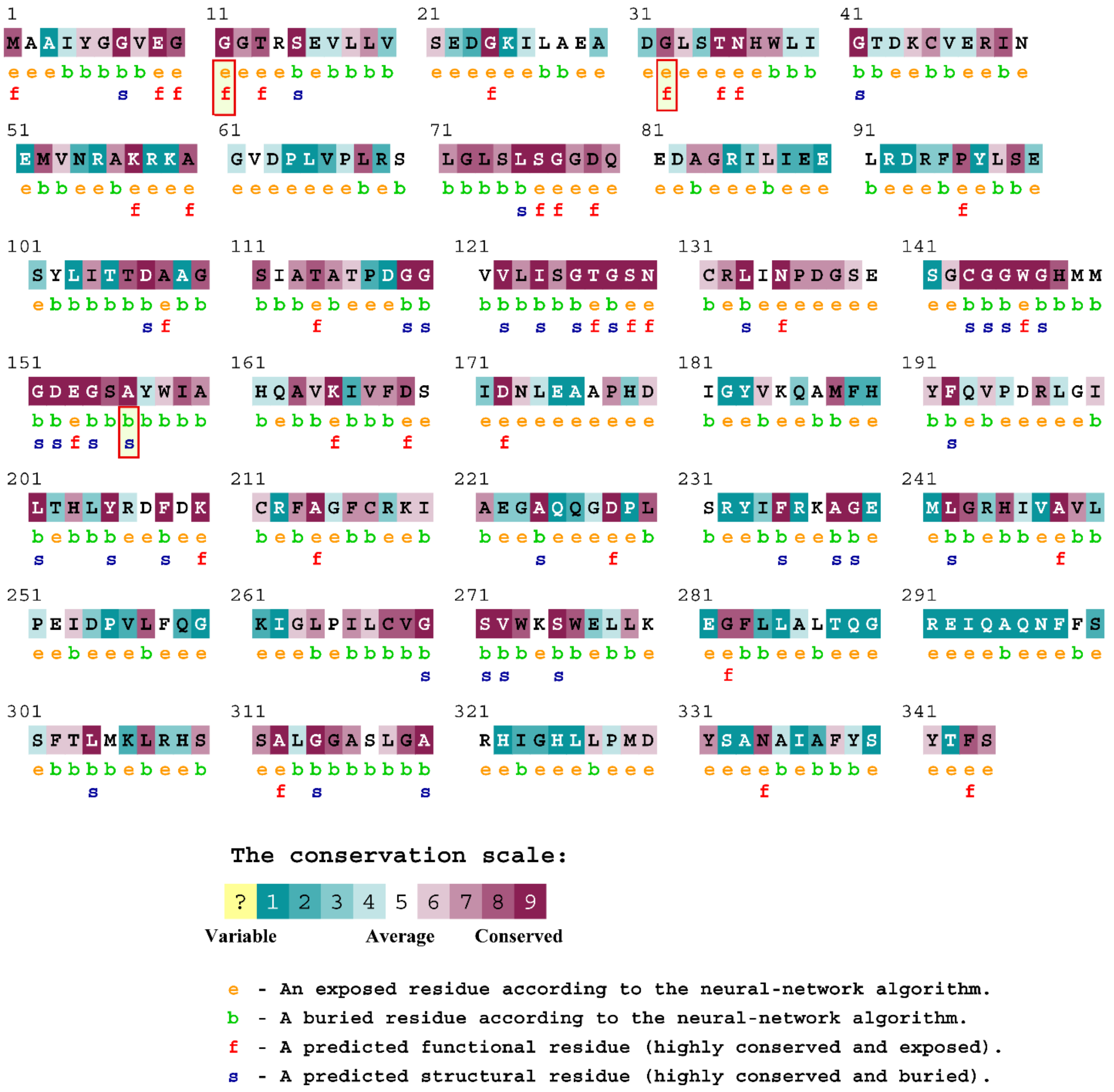

Figure S1. Analysis of Consurf tool representing the conservation profile of amino acids in NAGK. Identified variant positions are highlighted in red box.

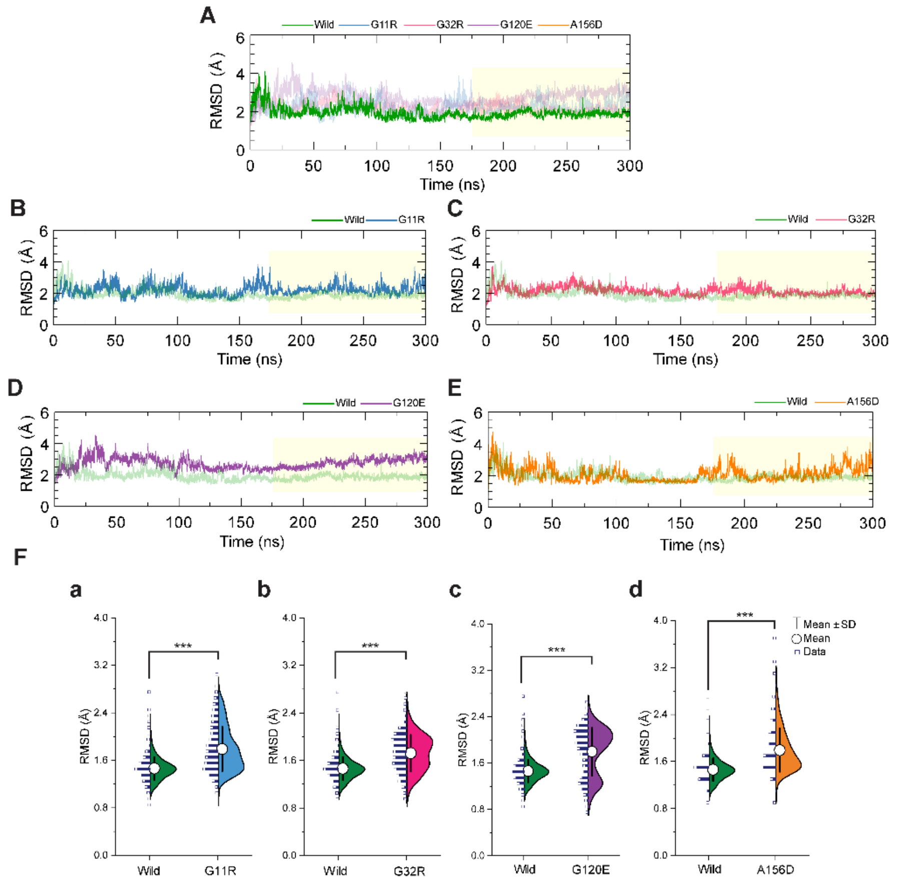

Figure S2. Evaluation of simulation stabilities. **(A)** Comparative root-mean-square deviations (RMSDs) analysis of C$_\alpha$ atoms for wild-type and NAGK variants, where green line demonstrates the wild-type, and the blue, pink, violet, and orange lines represent G11R, D32R, G120E, and A156D, respectively. RMSD plot of G11R **(B)**, G32R **(C)**, G120E (**D**), and A156D (**E**) variants as compared with NAGK wild-type. The last 125 ns of trajectories of systems, as highlighted by the yellow shade, were considered for further analysis. (F) Half violin plot describes probability density and mean difference in the RMSD distribution, based on the last 125 ns trajectories. The blue, pink, violet, and orange lines indicate G11R, G32R, G120E, and A156D, respectively, while green denotes wild-type. The annotations

represent statistically significant, denoting *** $p < 0.0001$. Two-tailed, equal-sample variance Student's t-tests were used to calculate the P values.

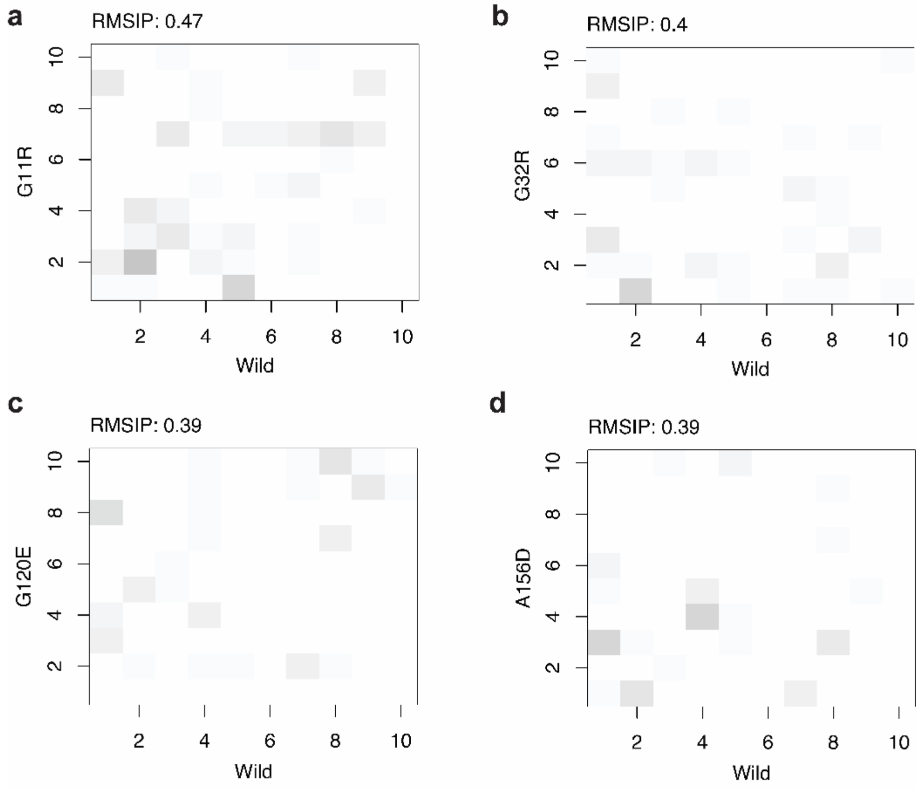

Figure S3. The RMSIP value was analyzed by overlapping matrix for the first ten principal components from sub-trajectories of wild and variants, including G11R (a), G32R (b), G120E (c), and A156D (d). The grey gradient visualizes the RMSIP values from white to black.

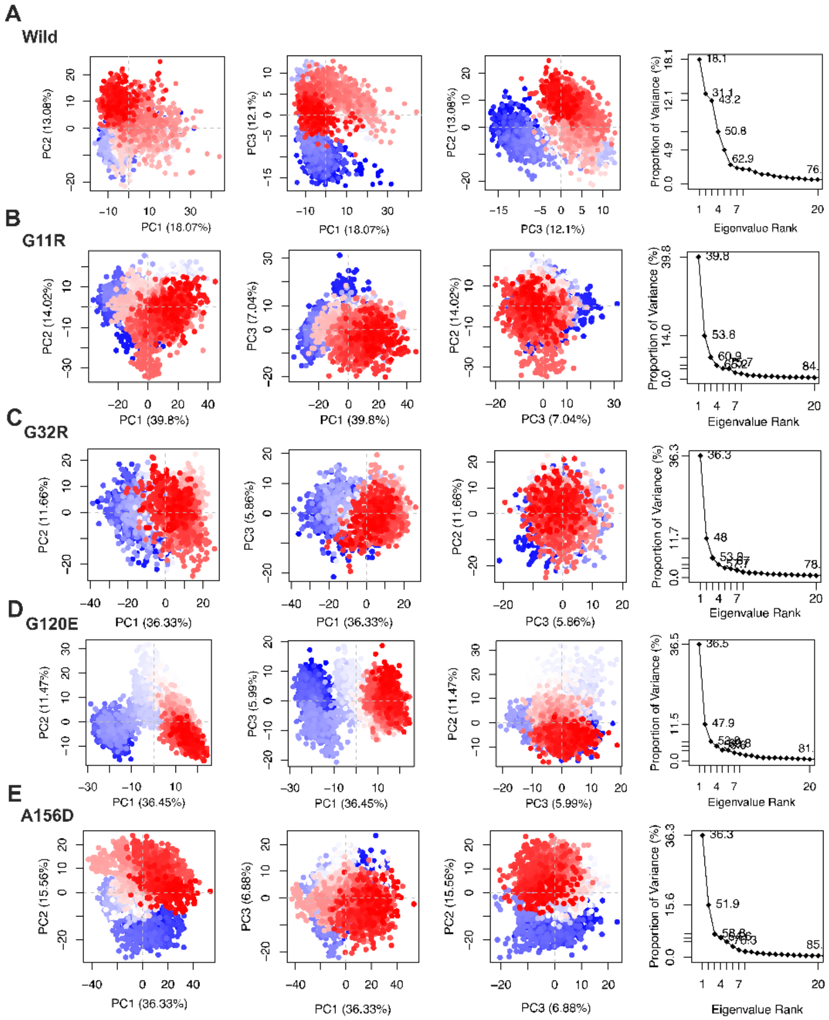

A
Wild
PC1 (18.07%)
PC2 (13.08%)
PC3 (12.1%)
Proportion of Variance (%)
Eigenvalue Rank
18.1
31.1
43.2
50.8
62.9
76
B
G11R
PC1 (39.8%)
PC2 (14.02%)
PC3 (7.04%)
39.8
53.8
60.9
84
C
G32R
PC1 (36.33%)
PC2 (11.66%)
PC3 (5.86%)
36.3
48
78
D
G120E
PC1 (36.45%)
PC2 (11.47%)
PC3 (5.99%)
36.5
47.9
81
E
A156D
PC1 (36.33%)
PC2 (15.56%)
PC3 (6.88%)
36.3
51.9
85

Figure S4. Principle Component Analysis (PCA) of NAGK dynamics in different systems, including wild (A), G11R (B), G32R (C), G120E (D), and A156D (E). The conformational distribution of each protein in a different system was captured on by the first three principal components, where a single protein conformer is represented by each dot with the simulation time, represented by a color-coded scale from blue to white to red. The variance captured by eigenvectors is also represented.

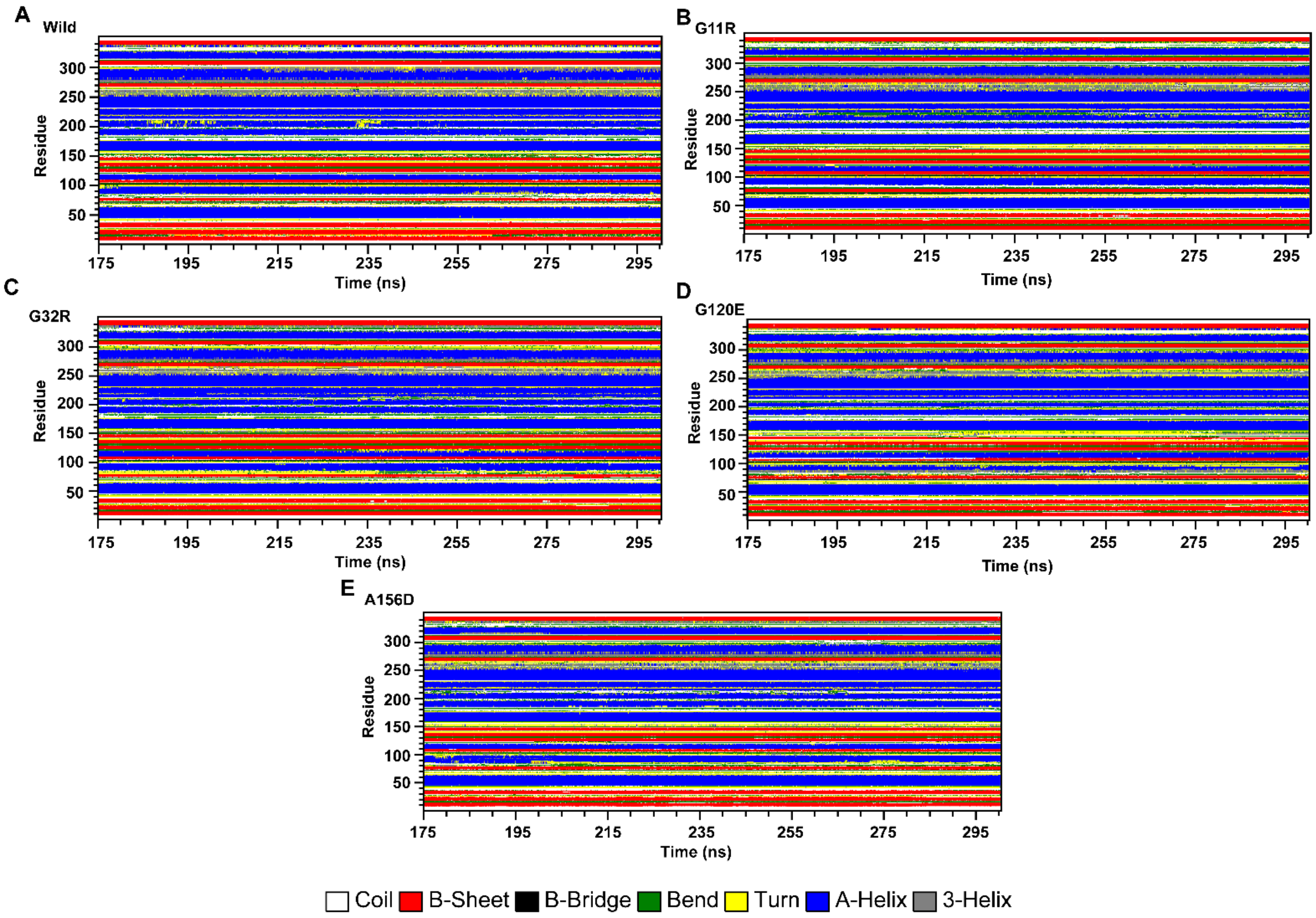

Figure S5. Variants induced changes in the secondary structure elements of NAGK. (A) Wild-type, (B) G11R, (C) G32R, (D) G120E and (E) A156D.

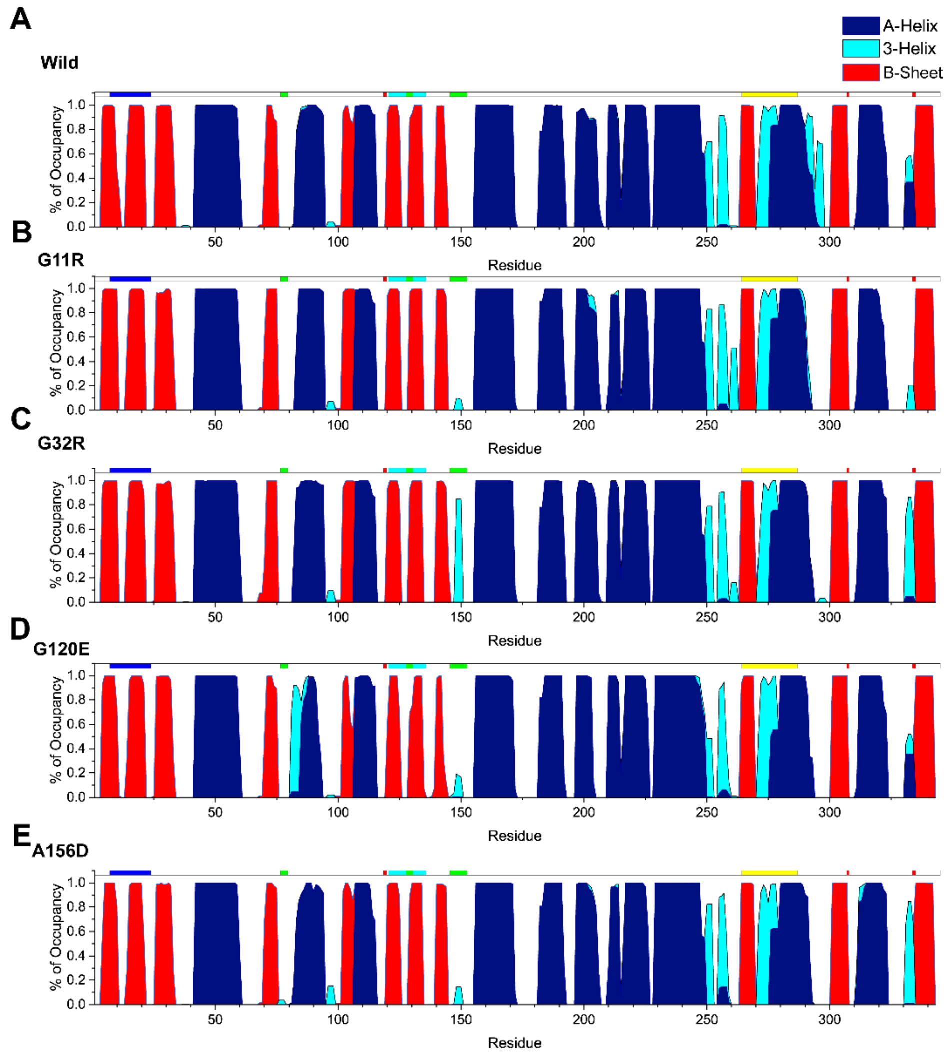

Figure S6. Residue-wise average secondary structural occupancies for wild (A), G11R (B), G32R (C), G120E (D), and A156D (E) structure, during the last 125 ns simulation. Here, dark blue color stands for beta-sheet and red color for alpha-helix conformation.
